# Surveillance of surgical site infections by *Pseudomonas aeruginosa* and strain characterization in Tanzanian hospitals does not provide proof for a role of hospital water plumbing systems in transmission

**DOI:** 10.1186/s13756-017-0216-x

**Published:** 2017-06-06

**Authors:** Nyambura Moremi, Heike Claus, Ulrich Vogel, Stephen E. Mshana

**Affiliations:** 1 Institute for Hygiene and Microbiology, University of Wuerzburg, Josef-Schneider-Street 2 / Building E1, 97080 Wuerzburg, Germany; 2 Department of Microbiology and Immunology, Catholic University of Health and Allied Sciences, Bugando, Mwanza, Tanzania

**Keywords:** *P. aeruginosa*, Surgical site infection, Water microbiology, Tanzania

## Abstract

**Background:**

The role of hospital water systems in the development of *Pseudomonas aeruginosa* (*P. aeruginosa*) surgical site infections (SSIs) in low-income countries is barely studied. This study characterized *P. aeruginosa* isolates from patients and water in order to establish possible epidemiological links.

**Methods:**

Between December 2014 and September 2015, rectal and wound swabs, and water samples were collected in the frame of active surveillance for SSIs in the two Tanzanian hospitals. Typing of *P. aeruginosa* was done by multi-locus sequence typing.

**Results:**

Of 930 enrolled patients, 536 were followed up, of whom 78 (14.6%, 95% CI; 11.6–17.5) developed SSIs. *P. aeruginosa* was found in eight (14%) of 57 investigated wounds*.* Of the 43 water sampling points, 29 were positive for *P. aeruginosa*. However, epidemiological links to wound infections were not confirmed. The *P. aeruginosa* carriage rate on admission was 0.9% (8/930). Of the 363 patients re-screened upon discharge, four (1.1%) possibly acquired *P. aeruginosa* during hospitalization. Wound infections of the three of the eight *P. aeruginosa* SSIs were caused by a strain of the same sequence type (ST) as the one from intestinal carriage. Isolates from patients were more resistant to antibiotics than water isolates.

**Conclusions:**

The *P. aeruginosa* SSI rate was low. There was no evidence for transmission from tap water. Not all *P. aeruginosa* SSI were proven to be endogenous, pointing to other routes of transmission.

## Background

*Pseudomonas aeruginosa (P. aeruginosa)* has emerged as an important opportunistic pathogen [1]. *P. aeruginosa* is mostly found in moist environments including hospital water systems [2, 3]. Its ability of forming biofilms contributes to its persistence in water system [4] hence a potential reservoir for *Pseudomonas* surgical site infections. Wound infections especially caused by multidrug-resistant *P. aeruginosa* strains are associated with increased morbidity and mortality [5].

Colonization of hospital water plumbing systems with *P. aeruginosa* has been shown to be an important source of the bacteria facilitating transmission to patients [6, 7]. Other sources such as contamination by *P. aeruginosa* of healthcare workers’ hands [8] and patient’s *P. aeruginosa* intestinal carriage [9] have been established to be other potential routes of transmission. The proportion of *P. aeruginosa* among other bacteria causing wound infections in Tanzania has been reported to be 16.3% (2014) at Muhimbili National Hospital [10] and between 27% (2014) and 40% (2016) at the Bugando Medical Centre (BMC) [11, 12]. In both hospitals, *P. aeruginosa* was found to contribute significantly to wound infections. Despite the fact that surgical site infections (SSIs) is among global burdens which requires priority [13], routine surveillance as an infection control measure [14] is not done in most low income countries.

In this study we conducted surveillance of SSIs at a Tanzanian regional and a tertiary hospital to assess the burden of SSI and to specifically link *P. aeruginosa* SSI to asymptomatic carriage and hospital water in order to determine the source.

## Methods

### Study design and setting

A prospective cohort study was conducted between December 2014 and September 2015 at Sekou Toure and BMC hospitals in the Mwanza region. Sekou Toure is a regional referral hospital with a bed capacity of 300. The BMC is a tertiary referral hospital for 10 out of 30 regions of Tanzania, which has a bed capacity of 1000 and serves about 18 million people. A total of 930 patients who were admitted for surgery (general surgery, obstetrics, gynaecology and orthopaedic) at the two hospitals within the study period were enrolled into the study after signing a written informed consent. Their socio-demographic information and medical history relevant to the study were recorded.

### Infection surveillance

Rectal swabs were taken using sterile Amies swabs (Mast Group Ltd., United Kingdom) within 48 h of admission (before surgery), and on discharge to assess *P. aeruginosa* carriage status. On admission carriage was defined as a positive screening culture within 48 h of being admitted to the hospital in absence of positive clinical specimen [15, 16]. On discharge carriage was defined as a positive screening culture when the patient was discharged from the hospital. Hospital acquired carriage was considered when a strain of *P. aeruginosa* was not detected upon admission screening or in case of acquisition of a strain of *P. aeruginosa* with a different sequence type (ST) during hospital stay on discharge.

Patients were followed up by either a surveillance doctor or a trained nurse after surgery to register signs and types of SSI according to NHSN definitions [15]. In case of clinical SSI, a surveillance doctor or a trained nurse took swabs for microbiological investigation. Surveillance doctor’s mobile phone number was given to discharged patients to notify the doctor in case they noted any signs or symptoms of SSI. The total surveillance period was until either a SSI became apparent or up to 30 days after being operated. Patients who underwent orthopaedic surgeries including foreign body implantation were followed-up for 90 days. Text messages were sent to patients every other day to remind them to notify a surveillance doctor when they noted any signs or symptoms of SSI.

### Water sampling

Sekou Toure hospital receives its water from a deep drilled well within the hospital compound which is locally chlorinated before being used, whereas BMC hospital receives water from Lake Victoria treated by a modern Capri-point Water Treatment Plant and therefore not locally chlorinated as a routine. The aim of this study was to investigate hospital water used routinely by staff and patients without applying any intervention so as to match recovered *P. aeruginosa* isolates with patients’ isolates.

Three water taps were identified for cold water sampling as per above explained purposes in each of the 11 wards where patients were enrolled. In addition, operating theatres and main water distribution points were sampled. In total 16 and 27 sampling points were defined in Sekou Toure and BMC hospitals, respectively. Water samples were collected as per DIN EN ISO 19458 (water sampling for microbiological analysis) monthly for up to 10 months in BMC and for four months in Sekou Toure hospital. Water sampling according to purposes A, B, and C was performed as outlined in the international standard EN/ISO 19458:2006 [17] with the aim of assessing the quality of water at the point of delivery to the hospital to rule out contamination from other sources outside hospital premises (purpose A), the quality of the waterlines supplying the taps (purpose B), and the possible contamination of the taps themselves (purpose C). The main difference between purposes A and B is the water volume discarded to flush the disinfectant before sampling, which was 10 L for purpose A and 1.5 L for purpose B. In contrast to purposes A and B, the sampling points were not disinfected for purpose C. A 125 ml-sampling bottle containing sodium thiosulfate (final concentration in the water sample: 20 mg/l) was used. At the Sekou Toure hospital sampling was solely conducted according to purpose C due to the aforementioned nature of water source.

A double-concentrated malachite base (Merck Millipore, Germany) was prepared and when cooled was supplemented with malachite-green oxalate solution (final concentration of 0.02 g/l). Malachite green broth enrichment was used to investigate the presence of *P. aeruginosa* in water [18], because filtration of water or alternative most probable number approaches required technical equipment not available on site.

### Isolation of *P. aeruginosa* from water

One hundred millilitres of the collected water were inoculated into the 250 ml glass bottles containing 100 ml of malachite-green broth (final concentration of 0.01gmalachite green oxalate /l) and incubated aerobically at 37 °C for 24 to a maximum of 72 h. In case of turbidity and/ or colour changes from green to yellow, 100 μl was sub-cultured onto blood (BD Difco, USA) and cetrimide (Merck Millipore, Germany) agars. The plates were incubated at 37 °C for 24 h. Yellowish-green colonies on cetrimide agar matching the oxidase positive colonies on blood agar were regarded positive for *P. aeruginosa*. Identification was confirmed by VITEK-MS (bioMérieux, France), because this was the method of choice also for the patient isolates.

### Analysis of *P. aeruginosa* from patients

Sterile cotton swabs (Mast Group Ltd., United Kingdom) were used to collect rectal and pus/wound swab from patients for carriage and infection purposes, respectively. Gram staining and culture of the pus/wound swab was performed in parallel. Pus/wound and rectal swabs were inoculated onto blood and MacConkey agars (BD Difco, USA), respectively, incubated at 37 °C, and examined for growth after 24–48 h. Oxidase test was performed to all non-lactose fermenting colonies. Oxidase-positive colonies were further analysed by VITEK-MS (bioMérieux, France).

All *P. aeruginosa* isolates were subjected to antimicrobial susceptibility testing using VITEK-2 system (bioMérieux, France) according to the manufacturer’s recommendations. Isolates with intermediate susceptibility were regarded as resistant in the analysis. The recommendations of EUCAST (http://www.eucast.org/clinical_breakpoints/) were applied for evaluation.

### Multilocus sequence typing

Multilocus sequence typing (MLST) using seven housekeeping genes was performed as previously described [19]. The PCR products were sequenced at GATC Biotech AG (Cologne, Germany). Sequence alignment and analysis was done using MegAlign software (DNASTAR Inc. USA) and the *P. aeruginosa* MLST website http://pubmlst.org/paeruginosa/was used to assign isolates to their respective sequence types (STs).

### Carbapenemase gene screening

All four *P. aeruginosa* isolates with either intermediate or resistant susceptibility to carbapenems were screened for metallo-beta lactamase genes (*bla*_IMP_, *bla*_VIM_ [20], *bla*_GIM_, *bla*_NDM_, *bla*_SIM_, *bla*_SPM_ and *bla*_OXA-48_) [21]as described previously.

### Data analysis

Data were analysed using STATA version 13 (STATA Corp LP, USA). Categorical variables were summarized as proportions and were analysed using the Pearson’s Chi-Square test or Fisher’s exact to test statistical differences among the various groups. The two-sample test of proportion was used to calculate 95% confidence interval (CI) and the Mann Whitney ranksum test was performed to compare medians. A *p*-value of less than 0.05 was considered statistically significant.

## Results

### Demographics

A total of 930 patients (57.8% female) were enrolled. The BMC tertiary hospital contributed to 64.9% (*n* = 604). The median age of the participants was 32.1 (range 2 months-83 years). Most patients came from Mwanza (61.9%, *n* = 576), Mara (10.1%, *n* = 94) and Shinyanga (8.5%, *n* = 79) regions. Of the 930 patients screened for *P. aeruginosa* carriage on admission, 363 were re-screened on discharge. After discharge follow-up was restricted to patients with mobile phones, therefore, 57.6% (536/930) of the enrolled patients were successfully followed-up. The median age (years) of followed-up patients was 26 (IQR: 18–42) while for those not followed-up was 31 (IQR: 23–48), *p* = 0.0001. Other socio-demographic parameters (sex, hospital, marital status, occupation etc) were equally distributed within the two groups.

### SSI rates, types and *P. aeruginosa* carriage

Of the 536 patients followed-up after discharge, 78 (14.6%, 95% CI; 11.6–17.5) developed SSI. The wounds of 57 patients were investigated microbiologically, of which 50 (87.7%) had significant bacterial growth and eight (14%) were positive for *P. aeruginosa*. All patients with *P. aeruginosa* SSI were classified as superficial incisional SSI (A1).

Of the 930 patients screened on admission, eight (0.9%) were found to be colonized with *P. aeruginosa* as demonstrated by rectal swabs. Of the 363 patients re-screened on discharge, seven (1.9%) were colonized with *P. aeruginosa*. Of those, four possibly acquired the strain during hospitalization and the remaining three patients were colonized upon admission and discharge.

### *P. aeruginosa* In the water distribution

Cold water samples were taken from taps located in wards as well as in the operating theatres. The mean (±standard deviation) water temperature was 26.2 (±0.4) and 25.8 (±0.8)°C at BMC and Sekou Toure hospitals, respectively. Twenty-two (81.5%) of the 27 water sampling points from BMC hospital were positive for *P. aeruginosa* throughout the study period; 11 (40.7%) were positive for *P. aeruginosa* at least twice (Table 1). At BMC hospital, sampling points were positive in the months December 2014 (*N* = 11), and January (*N* = 6), August (*N* = 6) and September (*N* = 15) 2015, resulting in 38 *P. aeruginosa* isolates. Seven (44%) of the 16 sampling points from Sekou Toure hospital were positive throughout the study period; only one sampling point was positive more than once resulting in ten isolates (Table 2).

**Table 1:** Sequence type distribution among *Pseudomonas aeruginosa* detected at 22 out of 27 sampling points at Bugando Medical Centre hospital

	Sampling point	Ward/ sampling point category	Sampling plan	Number of *P. aeruginosa* recovery from water taps in 10 months	Sequency type of *P. aeruginosa*
1	Main distribution	-	A	2 of 10	**381**, 2320^a^
2	OT changing room	Operating Theatre	B	2 of 10	**381**
3	OT2	Operating Theatre	C	3 of 10	**381**, 252, 2307^a^
4	OT3	Operating Theatre	C	1 of 10	**381**
5	OT5	Operating Theatre	C	1 of 10	**381**
6	OT sluice	Operating Theatre	C	1 of 10	**381**
7	LWOT	Maternity Operating Theatre	B	1 of 10	**381**
8	LW staff WC	Maternity	C	1 of 10	**381**
9	LW patient WC	Maternity	C	2 of 10	**381**, 834
10	C4 patient WC	Maternity	C	2 of 10	**381**, 641
11	C4 sluice	Maternity	C	1 of 10	2327^a^
12	E4 patient WC	Gynaecology	C	2 of 10	**381**, 2307^a^
13	E4 sluice	Gynaecology	C	1 of 10	2325^a^
14	J5 staff WC	Orthopaedic	B	4 of 10	**381**, 834, 2307^a^
15	J5 patient WC	Orthopaedic	C	1 of 10	2326^a^
16	C6 staff WC	General surgery	B	3 of 10	**381**, 834
17	C6 patient WC	General surgery	C	1 of 10	**381**
18	E8 Staff WC	Orthopaedic	B	1 of 10	**381**
19	E8 patient WC	Orthopaedic	C	3 of 10	**381**
20	C9 staff WC	General surgery	B	2 of 10	**381**
21	C9 patient WC	General surgery	C	1 of 10	**381**
22	C9 sluice	General surgery	C	2 of 10	**381**, 236

Key: WC: Water Closet (Toilet); ^a^New ST; bold letters indicate common clone

**Table 2:** Sequence type distribution among *Pseudomonas aeruginosa* detected at seven out of 16 total sampling points at Sekou Toure hospital

	Sampling point	Ward/ sampling point category	Sampling plan	Number of *P. aeruginosa*recovery from water taps in 4 months	Sequency type of *P. aeruginosa*
1	Main distribution	-	C	1 of 4	2307*
2	STGN station	Gynaecology	C	4 of 4	2307*
3	FW station	Female	C	1 of 4	252
4	MW2 patient WC	Male	C	1 of 4	316
5	LW station	Maternity	C	1 of 4	2307*
6	OT1	Operating theatre	C	1 of 4	2307*
7	OT2	Operating theatre	C	1 of 4	2307*

Key: WC: Water Closet (Toilet); ^a^New ST; bold letters indicate common clone

### Sequence types distribution

A total of 18 different sequence types (STs) was observed among 71 *P. aeruginosa* isolates of which eight were new STs. Ten STs occurred only once (Table 3). Of the eight patients with *P. aeruginosa* SSI, four from the BMC hospital harboured the multi-resistant ST235. Two of the four patients with SSI due to *P. aeruginosa* ST235 were treated in the same ward and developed SSI two days apart. Three patients with SSI harboured strains bearing the same STs as those in their intestines i.e. STs 235, 2309 and 2319 (Table 4). Three patients carried *P. aeruginosa* isolates that shared STs with isolates recovered from water taps of the wards they were admitted in (Table 4). As shown in Table 3, the overlap of STs of strains from patients and the water distribution was minimal, only STs 2307 and 252 were observed in both hospitals. ST2307 and ST381 were observed in 66.7% (8/12) and in 42.4% (25/59) of isolates from Sekou Toure and BMC hospital, respectively.

**Table 3:** Sequence type distribution among *Pseudomonas aeruginosa* isolates from Sekou Toure and Bugando Medical Centre hospitals

Sequence type	Sekou Toure hospital (*N* = 12)		BugandoMedical Centre hospital (*N* = 59)	
	Patients (2)	Water(10)	Patients (21)	Water (38)
2307^a^	-	8	**4**	**3**
2309^a^	2	-	-	-
2317^a^	-	-	1	-
2319^a^	-	-	4	-
2320^a^	-	-	-	1
2325^a^	-	-	-	1
2326^a^	-	-	-	1
2327^a^	-	-	-	1
235	-	-	6	-
236	-	-	-	1
244	-	-	1	-
252	-	1	-	1
316	-	1	-	-
381	-	-	**1**	**25**
399	-	-	3	-
553	-	-	1	-
641	-	-	-	1
834	-	-	-	3

Key: ^a^New ST; bold letters indicate sequence type identity shared by patients and water samples

**Table 4:** Possible transmission sources among 17 patients who carried and/or were infected with *Pseudomonas aeruginosa*

Patient ID	Age (years)	Sex	Hospital	Ward Category	Type of Surgery	Hospital stay (days)	P.a. Carriage at Admission	P.a. strain (ST)	P.a. Carriage at Discharge	P. a. strain (ST)	SSI with P. a.	P. a*.* strain (ST)	P. a. strain (ST) in admitting ward
70	55	M	Bugando	General surgery	Laparotomy	7	**Yes**	2319^a^	No	-	No	-	381, 834
93	67	M	Bugando	General surgery	Esophagotomy	2	**Yes**	**2319** ^a^	**Yes**	**2319** ^a^	**Yes**	**2319** ^a^	381, 834
528	26	M	Bugando	General surgery	Laparotomy	18	No	-	No	-	**Yes**	2317^a^	381, 834
532	27	M	Bugando	General surgery	Colostomy	6	No	-	**Yes**	553	No	-	381, 834
323	1	F	Bugando	General surgery	Fistulectomy	8	No	-	**Yes**	399	No	-	381, 236
436	47	M	Bugando	General surgery	Mastectomy	2	**Yes**	399	**Yes**	399	No	-	381, 236
477	63	F	Bugando	General surgery	Mastectomy	2	**Yes**	**381**	No	-	No	-	**381,** 236
GN001	58	F	Bugando	Gynaecology	Laparotomy	10	**Yes**	**2307** ^a^	**Yes**	**2307** ^a^	No	-	381, **2307** ^a^, 2325^a^
GN002	49	F	Bugando	Gynaecology	Myomectomy	10	No	-	**Yes**	**2307** ^a^	No	-	381, **2307** ^a^, 2325^a^
GN003	45	F	Bugando	Gynaecology	Laparotomy	4	**Yes**	**2307** ^a^	No	-	No	-	381, **2307,** ^a^ 2325^a^
GN026	33	F	Bugando	Gynaecology	Laparotomy	8	No	-	No	-	**Yes**	244	381, 2307^a^, 2325^a^
33	27	M	Bugando	Orthopaedic	ORIF	23	No	-	No	-	**Yes**	235	381
41	28	M	Bugando	Orthopaedic	ORIF	21	No	-	No	-	**Yes**	235	381
245	83	M	Bugando	Orthopaedic	ORIF	10	**Yes**	235	No	-	No	-	381
11	54	F	Bugando	Orthopaedic	ORIF	33	No	-	No	-	**Yes**	235	381, 834, 2307^a^, 2326^a^
LW028	31	F	Bugando	Obstetrics	Csection	3	**Yes**	**235**	No	-	**Yes**	**235**	381, 834
ST098	30	F	Sekou Toure	Obstetrics	Csection	4	No	-	**Yes**	2309*	**Yes**	**2309** ^a^	2307^a^

Key: P.a; *Pseudomonas aeruginosa*; SSI; Surgical site infection; ORIF; Open Reduction Internal Fixation; Csection; Caesarean Section; ^a^New sequence type (ST); bold numbers indicate shared sequence type identity between carried and SSI *P. aeruginosa* or between carried *P. aeruginosa* and *P. aeruginosa* from water samples in the same ward. All 17 patients were followed-up for SSI

### Antimicrobial susceptibility

Fifty-six *P. aeruginosa* isolates were analysed of which 17 were non-repetitive isolates from patients and 39 from water. Only one strain per sequence type (ST) per patient and one strain per ST per sampling point were included in this analysis. All clinical and water isolates were resistant to aztreonam (Table 5). Of patients’ isolates, 41.2% (7/17), 35.3% (6/17) and 17.7% (3/17) were resistant to piperacillin-tazobactam, ceftazidime and meropenem/imipenem, respectively. Higher resistance rates were observed in patients in comparison to water isolates for piperacillin-tazobactam (*p* = 0.001), ceftazidime (*p* < 0.001) and amikacin (*p* = 0.0004). Fosfomycin resistance was significantly more frequent in water isolates than in clinical isolates (61.5% vs. 17.7%, *p* = 0.001) (Table 5).

**Table 5:** Resistance rates of *Pseudomonas aeruginosa* isolates from patients and water

Antimicrobial agent	Patients isolates (17)	Water isolates (39)	*P* value
	N (%)	N (%)	
Amikacin	5 (29.4)	0 (0)	0.0004
Aztreonam	17 (100)	39 (100)	-
Cefepime	1 (5.9)	0 (0)	0.063
Ceftazidime	6 (35.3)	0 (0)	<0.001
Ciprofloxacin	5 (29.4)	6 (15.4)	0.112
Colistin	0 (0)	0 (0)	-
Ertapenem	3 (17.7)	1 (2.6)	0.0256
Fosfomycin	3 (17.7)	24 (61.5)	0.001
Gentamicin	5 (29.4)	5 (12.8)	0.06
Imipinem	3 (17.7)	1 (2.6)	0.0219
Meropenem	3 (17.7)	0 (0)	0.0035
Piperacillin	8 (47.1)	7 (18.0)	0.012
Piperacillin-tazobactam	7 (41.2)	1 (2.6)	0.001
Tobramycin	5 (29.4)	2 (5.1)	0.005

All four isolates with reduced susceptibility to carbapenems were screened for carbapenemase genes, of which none of them tested positive.

## Discussion

In this study the rate of *P. aeruginosa* SSI was low and accounted for a minor proportion of all SSIs. One reason for this might be the low intestinal carriage rate on admission imposing a low risk of endogenous infection [9]. Despite the low number of patients with *P. aeruginosa* SSI, this study confirmed intestinal carriage as a source of infection in three patients based on MLST typing. As explained previously [22], personal hygiene has been found to contribute to endogenous transmission. This is further supported in the current study by the fact that the three patients with an evidence of endogenous source developed infection after being discharged from the hospital; explaining the possibility of poor hygiene at home during the change of dressing.

In the current study the difference of *P. aeruginosa* carriage rates upon admission and discharge was not statistically significant. The relative low rate observed on the discharge could be explained by the low yield of a single-time swabbing compared to multiple swabbing [9]. However, as documented previously [23] regarding hospital acquisition of *P. aeruginosa*, four patients who were negative on admission were found to be colonized upon discharge, indicating possible hospital acquisition of *P. aeruginosa*.

Out of eight patients with *P. aeruginosa* SSI, four were found to belong to ST235, a multi-resistant clone, which is widely distributed in European [24, 25] and Asian countries [26, 27]. Unlike previous reports on this international high-risk clone [25, 28, 29], carbapenemase genes such as *bla*_VIM-2_ were not identified by PCR. Interestingly, two of the four patients with *P. aeruginosa* ST235 SSI were spatio-temporally linked; pointing to the possibility of a common source in the ward.

Although more than 80% of the sampled water taps at BMC hospital were at least once positive for *P. aeruginosa* during the observation period, no clear linkage to *P. aeruginosa* SSI was established in contrast to what has been reported previously [6]. This observation could be explained by the fact that, the taps were found to be *P. aeruginosa* free amidst the surveillance period following the intervention such as local chlorination made by the BMC hospital infection control team after seeing preliminary sampling results. This could have affected the link of *P. aeruginosa* SSI to water system because during the intervention period patients were at risk of getting *P. aeruginosa* SSI but the exogenous risk (water system colonization) was absent.

Another reason might be the possibility of low bacterial loads. Due to the technique employed in this study, only the presence of *P. aeruginosa* was detected, but not the quantity. Although the current study could not establish the association between water system and *P. aeruginosa* SSI, two sequence types (ST381 and ST2307) were shared between patient’s carriage and water system; underscoring the possible role of water system in cross-transmission of *Pseudomonas* [30]. Despite established evidence that *P. aeruginosa* contamination of wastewater systems such as toilets and shower sinks [31] might also serve as sources of infection, wastewater systems were not analysed for *P. aeruginosa* in this study.

## Conclusions

To the best of our knowledge this is one of the largest studies on the prevalence of *P. aeruginosa* induced SSI in Africa. Post-discharge surveillance was effective due to the use of text message recalls. Although the rate of *P. aeruginosa* SSI was low, endogenous sources appeared to be a more probable source of transmission than the hospital water system. Multi-resistance of *P. aeruginosa* to clinically used antibiotics is an issue which needs to be taken into account.

## Abbreviations

BMC: Bugando Medical Centre
DIN: Deutsches Institut für Normung (German Institute for Standardization)
EN: European Committee for Standardization
EUCAST: European Committee on Antimicrobial Susceptibility Testing
ISO: International Organization for Standardization
MLST: Multilocus sequence typing
NHSN: National Healthcare Safety Network
SSI: surgical site infection
ST: Sequence type
